# Concordance between self-report and medical records of preventive healthcare delivery among a sample of disadvantaged patients from four aboriginal community controlled health services

**DOI:** 10.1186/s12913-019-3930-7

**Published:** 2019-02-08

**Authors:** Natasha Noble, Christine Paul, Justin Walsh, Kylie Wyndham, Sue Wilson, Jessica Stewart

**Affiliations:** 1 Priority Research Centre for Health Behaviour, School of Medicine and Public Health, University of Newcastle, Callaghan, NSW 2308 Australia; 2 The Hunter Medical Research Institute, New Lambton Heights, NSW 2305 Australia; 3 Bulgarr Ngaru Medical Aboriginal Corporation, Richmond Valley Clinic, 153 – 157 Canterbury St, Casino, NSW 2470 Australia; 4 Durri Aboriginal Corporation Medical Service, 15-19 York Lane, Kempsey, NSW 2440 Australia; 5 NSW Department of Family & Community Services- Business Services, 219-241 Cleveland Street, Redfern, NSW 2016 Australia

**Keywords:** Aboriginal, Indigenous, Self-report, Medical record, Agreement, Concordance, Preventive care

## Abstract

**Background:**

This cross-sectional study aimed to explore, among a sample of patients attending one of four Aboriginal Health Services (ACCHSs), the degree of concordance between self-report and medical records for whether screening for key healthcare items had ever been undertaken, or had been undertaken within the recommended timeframe.

**Methods:**

Across the four ACCHSs, a convenience sample of 109 patients was recruited. Patients completed a self-report computer survey assessing when they last had preventive care items undertaken at the service. ACCHS staff completed a medical record audit for matching items. The degree of concordance (i.e. the percentage of cases in which self-reports matched responses from the medical record) was calculated.

**Results:**

Concordance was relatively high for items including assessment of Body Mass Index and blood pressure, but was substantially lower for items including assessment of waist circumference, alcohol intake, physical activity, and diet.

**Conclusions:**

Reliance on either patient self-report or medical record data for assessing the level of preventive care service delivery by ACCHSs requires caution. Efforts to improve documentation of some preventive care delivery in medical records are needed. These findings are likely to also apply to patients in other general practice settings.

**Electronic supplementary material:**

The online version of this article (10.1186/s12913-019-3930-7) contains supplementary material, which is available to authorized users.

## Background

For many disadvantaged and indigenous groups, such as the Aboriginal and Torres Strait Islander peoples of Australia, the prevalence of risk factors for chronic disease, such as high blood pressure and smoking, is disproportionately high [1]. Reductions in such risks factors are needed in order for the health of disadvantaged and indigenous groups to achieve equality with the general population [2]. Primary care has a key role to play in assisting patients to improve their health risk status [3]. For Aboriginal Australians, a large proportion of the population receive primary and preventive care from an Aboriginal Community Controlled Health Service (ACCHS) [4]. ACCHSs are similar to other primary health care services, but focus on providing wholistic and culturally appropriate services to the Aboriginal communities they serve [5].

Evaluation of the delivery of healthcare is essential for improving the quality of health services, as such assessments can identify gaps in care. The provision of preventive care can be evaluated through medical records, or patient or provider self-report. However, the best source of information for assessing preventive care delivery remains uncertain [6]. Patient recall can be poor for a range of clinical interactions, [7] yet it remains an important indicator of the effective receipt of healthcare. Patients must be able to recall healthcare advice in order to adhere to the advice after a consultation [8]. Self-report can sometimes be less costly and more convenient than extracting data from medical record review [6]. Medical records can also have variable accuracy as a measure of healthcare delivery, with missing data a particular concern [9]. Despite no single validated measure (patient recall, provider recall or medical record audit) having a high and consistent level of accuracy, these measures are often used as indicators of healthcare delivery or service performance for quality improvement and other purposes [10]. Therefore, it is important to explore the level of concordance in such assessments across populations and health settings.

Relatively little attention has been directed towards assessing measures of healthcare delivery among highly vulnerable or disadvantaged groups, where rates of comorbidity are often high, [11, 12] and factors such as poor health literacy have the potential to influence both delivery and receipt of healthcare [11, 13]. It is particularly important to explore concordance between measures of care among patient groups who experience socioeconomic disadvantage and high levels of comorbidity, such as Indigenous Australians [12, 14]. For Indigenous Australians, the regular assessment of a number of key preventive health items within primary care is recommended. Risk factors including: smoking status, Body Mass Index (BMI), waist circumference, physical activity, alcohol consumption, diet, blood pressure, and cholesterol, should be assessed opportunistically, [15] and every 12 months as part of an annual comprehensive health assessment (Medicare item 715) [16].

### Aims

The aims of this study were to describe, among patients attending one of four ACCHSs, the degree of concordance between self-report and medical records for: a) whether key preventive healthcare items had ever been undertaken; and b) whether key preventive healthcare items had been undertaken within the recommended timeframe (the last 12 months).

## Methods

### Study design and setting

Data was collected as part of a larger ‘Chronic Care Service Enhancement Project’, involving a collaboration between ACCHSs from across NSW, the Centre for Aboriginal Health (NSW Ministry of Health), and the University of Newcastle. The larger project involved the collaborative implementation and evaluation of strategies to improve preventive and chronic disease care provided by ACCHSs in NSW. One component of the project examined the quality of data recorded by ACCHSs about care provided to patients. The level of accuracy of ACCHS medical record data was assessed by comparing data on matching clinical items collected from different sources including patient self-report and clinic medical records. Four ACCHSs agreed to take part in the data quality assessment, and only the results of this component of the larger study are reported here. The four ACCHSs were located in major cities (1 service), inner regional (2 services) and remote areas (1 service) of NSW [17]. The size of the services ranged from approximately 500 active patients (those who had visited the service at least once in the last 2 years), up to approximately 1700 active patients. All four ACCHSs used electronic Patient Information Management Systems for data recording and storage. Data collection was staggered across services, and occurred over several months (from two to six months) at each service during 2013 and 2014. Ethics approval for this study was obtained from the Aboriginal Health & Medical Research Council (AH&MRC) Ethics Committee (approval number 863/12) and the University of Newcastle (UoN) Human Research Ethics Committee (H-2012-0100).

### Participants and procedure

The four participating ACCHSs were asked to collect data about matching clinical items from consenting patients and the patient’s medical record. Staff including reception or an Aboriginal Health Worker at participating ACCHSs invited a convenience sample of approximately 30 patients per site to complete a touchscreen computer survey. Services determined which member of staff was suitable and had time to undertake recruitment. Patients were aged ≥18 yrs. Staff were asked to include a range of ages, gender, and patients with and without chronic disease. Patients attending one of the four ACCHSs for a GP appointment were asked to complete the survey immediately after their appointment. Written informed consent for survey completion, and to conduct an audit of the patient’s medical record, was sought from all participants. The survey asked patients to self-report when they were last screened for key health risk factors including: BMI, waist circumference, blood pressure, smoking status, alcohol intake, physical activity and diet, and when they last had preventive healthcare items including an annual Health Assessment (HA; Medicare item 715) and a Diabetes Care Plan (DCP; for patients with diabetes) undertaken by a healthcare provider at the service. A staff member undertook a manual search of consenting patients’ medical records and completed a standardised data sheet containing items matching those collected by the patient survey. For each item, staff were asked to indicate whether it had ever been recorded since the patient first attended the service (yes/no) and if so, the date of last assessment. No strict criteria were provided to determine whether a HA or DCP had been completed, and this may have been based on a completed template, or billed item number in the medical record. De-identified data was provided by each site to the research team.

### Derived variables

The degree of concordance between self-report and medical records was explored separately for two comparisons: a) whether key preventive healthcare items had ***ever*** been undertaken; and b) whether key preventive healthcare items had been ***undertaken within the recommended timeframe*** (the last 12 months).

For comparison a) whether an item had ***ever*** been undertaken, self-report variables were recoded to a binomial response (yes/no). All self-report response options indicating that the healthcare item had been undertaken (response options “≤12 m ago” or “> 12 m ago”) were coded as “yes”. All other responses, including “not sure”, “never”, and any missing responses, were coded as “no”. The medical record audit included a “yes” or “no” response for whether each item had been undertaken. Any blank/missing records in the medical record audit were coded as “no”.

For comparison b) whether an item had been ***undertaken within the recommended timeframe*** (the last 12 months), self-report variables were recoded to a binomial response. A response indicating that the healthcare item had been undertaken within the last 12 months (response option “≤12 m ago”) was coded as “yes”. All other responses were coded as “no” (including “> 12 m ago”, “never”, “not sure” and any missing values). Medical record dates for when an item was last undertaken were compared against the date of survey completion. Items that had been recorded within 12 months were coded as “yes”. Items which had been recorded more than 12 months before the date of survey completion were coded as “no”. Items that were not recorded in the medical record, or where the date was missing, were also coded as “no”. Twelve months was selected as it represents the annual screening interval recommended for the healthcare items included in the survey [15].

### Analysis

As there is no accepted gold standard criterion for evaluating the accuracy of healthcare performance measures, [18] the level of agreement between self-reported healthcare receipt and medical records was evaluated as concordance, defined as the percentage of cases in which self-reports matched responses from the medical record [18]. A sample size of 100 allowed the outcomes to be assessed with a 10% level of precision, assuming a prevalence rate of 50% and 95% level of confidence. Participants who self-reported that they had never smoked were not asked when their smoking status had been assessed at the medical service. Therefore only current and ex-smokers were included in the analysis of concordance about smoking status assessment. Similarly, only participants who self-reported having diabetes were asked about whether and how long ago they received a DCP and were included in the concordance analysis for a DCP.

## Results

### Sample

A total of 109 participants from four services (between 21 and 31 patients per service) had self-report survey and medical record audit data available for analysis. As this was a convenience sample, the consent rate was not recorded. Just over half of the sample were female (51%). Ninety-five percent of the sample identified as Aboriginal, Torres Strait or both Aboriginal and Torres Strait Islander Australians. The majority of the sample were aged 30–59 years (60%). The sample were regular patients, with almost half having visited their service at least seven times in the last 12 months, and approximately 95% of the sample having attended their service within the last 3 months. Almost all patients in the sample had one or more chronic disease (ranging from 78 to 95% across the four ACCHSs), most commonly diabetes and heart disease. Table 1 presents an overview of the number of service visits in the last year, and the prevalence of chronic disease, across the sample.

**Table 1:** Sample self-reported number of visits to the service in the last 12 months and prevalence of chronic disease

Sample characteristic	Prevalence *n* (%)
Number of visits to the service in the last 12 months	
1–3	12 (11%)
4–6	35 (32%)
7–9	15 (14%)
10 or more	36 (33%)
Not sure	11 (10%)
Self-reported chronic disease	
Diabetes	49 (45%)
Heart disease	26 (24%)
Kidney disease	12 (11%)
Depression	25 (23%)
Arthritis	20 (18%)
Cancer	2 (2%)
Other	31 (28%)
Not sure	3 (3%)

#### Aim a: To describe the degree of concordance between self-report and medical records for whether healthcare items had ever been undertaken

**Fig. 1:**
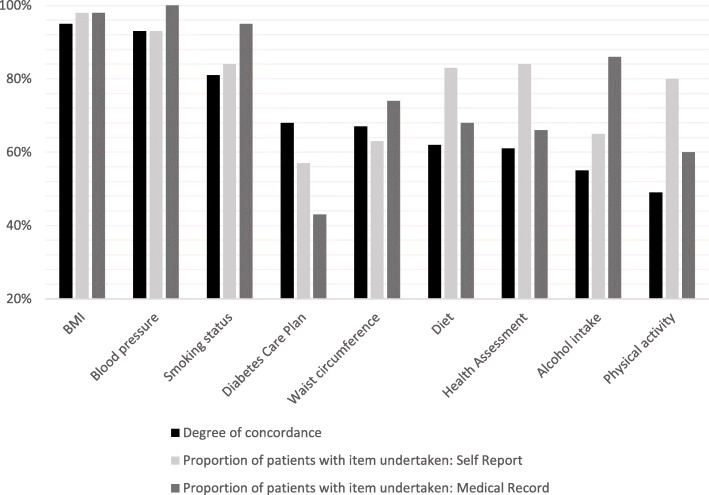
The degree of concordance between self-report and medical record data for whether healthcare items had ever been undertaken (from when the patient first attended the service), and proportion of patients for whom the healthcare items had ever been undertaken (from when the patient first attended the service) according to the two data sources [data collected 2013–14 from NSW]

The degree of concordance between self-report and medical records as to whether preventive healthcare items had ever been undertaken are presented in Fig. 1. Figure 1 also shows the proportion of patients for whom the item had ever been undertaken according to the two sources of data. More detailed data (including agreement, disagreement and missing records) are presented in Additional file 1 (Table S1).

The degree of concordance between self-report and medical records for whether BMI, blood pressure and smoking status had ever been screened was relatively high (> 80%). The degree of concordance between the two data sources varied from between 60 and 80% for whether patients had ever had their waist circumference or diet assessed, or had completed a HA or DCP. Concordance was < 60% for whether patients had ever had their alcohol intake or physical activity levels assessed.

#### Aim b: To describe the degree of concordance between self-report and medical records for whether healthcare items had been undertaken within the last 12 months

**Fig. 2:**
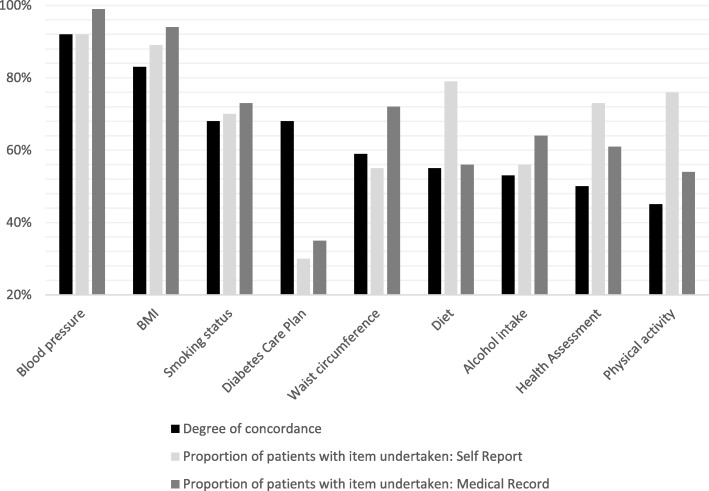
The degree of concordance between self-report and medical record data for whether healthcare items had been undertaken within the last 12 months, and proportion of patients for whom the healthcare items had been undertaken within this timeframe according to the two data sources [data collected 2013–14 from NSW]

Figure 2 presents the degree of concordance between self-report and medical records as to whether preventive healthcare items had been undertaken within the last 12 months. Detailed data (including agreement, disagreement and missing records) are also presented in Additional file 1 (Table S2).

The degree of concordance between data sources for whether preventive care items had been undertaken within the last 12 months was lower than for whether items had ever been undertaken (Aim a) across all healthcare items, except for the receipt of a DCP. Concordance between the two data sources for whether items had been undertaken within the last 12 months was above 80% for BMI and blood pressure, but was < 60% for screening of waist circumference, alcohol intake, physical activity, diet, and for completion of an annual HA.

## Discussion

This study is the first to compare self-report and medical record data for the provision of preventive healthcare among a sample of ACCHS patients attending one of four services in NSW. The degree of concordance between patient self-report and medical records was high for some items, including BMI and blood pressure, but substantially lower for other items such as alcohol intake, physical activity and diet assessment. The reliability of the two data sources appears to differ across healthcare items, and it is not clear which data source should be considered to be more accurate.

### Preventive care items where medical record appears to be more accurate

For items where the medical record indicated that a higher proportion of the sample had been assessed compared to self-report, it is probable that the medical record is the more accurate data source, given it is highly unlikely that these items would have been fabricated in the medical record [19]. These included the assessment of waist circumference, blood pressure, smoking status, and alcohol intake.

### Preventive care items where self-report appears to be more accurate

Conversely, for items where the self-reported prevalence of assessment was higher than according to medical records, it is likely that patient self-report is the more accurate data source. In these cases, the discrepancy may be due to a failure of the healthcare provider to record or update items in the patient’s medical record [20]. These items included assessment of physical activity and diet, and completion of an annual HA and DCP. However, for the latter two items, the higher prevalence according to self-report could be due to patients not being clear about what these specific items consist of, and thus not distinguishing these from more general care or general advice about health/ diabetes [18].

### Reasons for discrepancies between self-report and medical records

The discrepancies between self-report and medical records are likely to be due to either errors in patient recall or problems with medical record data recording or extraction. The high degree of concordance between assessment of risk factors including BMI and blood pressure is likely due to high rates of documentation of these activities in medical records, together with good patient recall. Activities such as having height or weight measured for BMI assessment, or use of a blood pressure cuff to assess blood pressure, are distinct procedures, for which patient self-report is likely to accurately reflect clinical practice [18].

Lower concordance for physical activity and diet screening appears to be due to a failure to document these activities in the medical record, with substantially more patients reporting that these had been assessed than compared to their medical record. This could be the result of a lower perceived priority of these health risks by healthcare providers, and subsequent failure to record these, or because of difficulties associated with the ability of clinical information systems to record such information. The poor concordance for smoking status and alcohol intake, where patients were less likely to report these as having been assessed than compared to documentation in their medical record, could further be due to the sensitive nature of these health behaviours [21]. Current smokers, or those drinking alcohol at higher than recommended levels, may be prone to disregard or underreport the evaluation of these behaviours. In addition, healthcare provider sensitivity about raising such issues [22, 23] may result in poorer patient recall. Given the high underlying rates of smoking and harmful alcohol use among many Aboriginal Australians, [2] this is a particular concern, as patients are subsequently also unlikely to recall and follow advice provided about smoking or reducing alcohol intake.

Given the unique setting of this study, there are limited data for direct comparison. In a study which compared patient and General Practitioner agreement about screening and risk status among 141 Aboriginal patients attending one ACCHS, there was reasonable agreement regarding smoking status, but poor agreement for alcohol consumption risk status, physical activity risk status, and for whether patients had been screened within the recommended timeframes for diabetes and blood pressure [24]. In two large USA studies, patient self-report and medical record data agreement for the receipt of risk factor counselling were broadly similar to the levels of agreement reported in the current study for physical activity and diet, but lower for smoking status. Levels of agreement ranged from 53% [6]-57% [21] for receipt of counselling on physical activity (compared to 49% concordance in the current study), 54% [6]- 60% [21] for diet (compared to 62% concordance in the current study), while agreement for the receipt of smoking cessation counselling was only 41% [6] (compared to 81% concordance in the current study). However, such findings are not directly comparable as the current study examined assessment of risk factors rather than the receipt of behavioural counselling. In the USA studies, the authors suggest inconsistent physician recording of counselling interventions is due to time pressures, [21] the result of such care not being financially reimbursed, [6, 21] and because of physician’s undervaluing the ‘talking’ aspects of medical care [21]. These factors are also likely to apply to assessment of healthcare items within the ACCHS setting, and indeed to general practice in Australia more broadly.

### Limitations

This study has a number of important limitations including the small sample size. The convenience nature of the sample may also limit the generalisability of the study findings to other ACCHSs. The sample included regular/frequently attending patients, who will have more opportunities for receiving preventive care than less regular patients, as well as greater potential for confusion in recalling timeframes- particularly for verbally delivered care, such as the assessment of diet or physical activity. The amount of missing data for some items was quite large, in particular for medical records of physical activity and diet assessment (see Additional file 1: Tables S1 and S2). As missing records were recoded as “no” for having ever/been assessed in the last 12 months, the amount of missing data will have affected the degree of concordance for these items. Unfortunately, BMI and waist circumference assessment data from one site were also lost and not able to be included in analysis, and comparisons of smoking status assessment and completion of a DCP were drawn from smaller samples of current or ex-smokers (*n* = 79), and those with self-reported diabetes (*n* = 37) respectively. Finally, self-report is subject to biases such as recall and social desirability, [25–27] as well as telescoping, where events are recalled as having occurred more recently than in reality [28]. The medical record audits were not subject to any cross-checking or inter-rater reliability measures and staff experience and training may have varied across sites.

### Implications for practice

Caution is required when relying on either self-report or medical record data alone to determine levels of preventive care service delivery in the ACCHS setting, as in other general practice settings [6]. This is particularly the case for whether items have been undertaken within recommended timeframes. Service providers and researchers need to be aware of the potential degree of error associated with either data source. While self-report and medical records appear to be relatively accurate for determining whether patient BMI, blood pressure or smoking status screening have been undertaken within the recommended timeframe, there is likely to be substantial under- or over-reporting of delivery of other preventive care items including alcohol intake, physical activity, diet, and completion of an annual HA or DCP, according to the data source used. While there are some unique considerations within the ACCHS setting, for example difficulties in accurately assessing alcohol intake which tends to be characterised by heavy but infrequent, episodic drinking, [29] the findings of this study are also likely to be applicable to other Australian general practice settings more broadly.

In order to obtain a more accurate reflection of the delivery of preventive care, multiple sources of data may need to be used [18, 21]. This is of concern given that many ACCHSs rely on medical record data extraction for reporting of national Key Performance Indicators (nKPIs), which are used nationally to monitor health outcomes for Aboriginal and Torres Strait Islander Australians [30]. nKPIs currently include smoking status, alcohol consumption, BMI and annual HA [30]. A number of quality improvement initiatives in Australian Aboriginal primary care have recognised the need to improve data collection processes, information systems, and documentation of delivered services [20, 31, 32]. A systematic review examining the quality of electronic patient records in primary care noted that, overall, lifestyle data were generally of lower quality than prescribing data [33]. Results of the current study reiterate the need to improve medical record documentation of preventive care, in particular for physical activity and diet. This might include staff training and the development of more user-friendly software, and/or automated system prompts to complete blank fields. Specific strategies to help improve both delivery and patient recall of smoking and alcohol risk assessment, and the receipt of behavioural counselling for these health risks, are also a high priority.

## Conclusion

Service providers and researchers need to be aware of the potential degree of error associated with relying on either self-report or medical record data to determine levels of preventive care service delivery in the ACCHS and other general practice settings. Self-report and medical records appear to be relatively accurate for determining whether patient BMI, blood pressure or smoking status screening have been undertaken within the recommended timeframe, however there is likely to be substantial under- or over-reporting of delivery of other preventive care items including alcohol intake, physical activity, diet, and completion of an annual HA or DCP, according to the data source used. Efforts are needed to improve medical record documentation of preventive care delivery, in particular for physical activity and diet. Similarly, strategies to improve patient recall of preventive care advice regarding smoking and alcohol intake are required.

## Additional file

Additional file 1: **Table S1.** and **Table S2**. Description of data: Detailed concordance data (including agreement, disagreement and missing records) for comparison a) whether an item had ***ever*** been undertaken (Table S1) and for comparison b) whether an item had been ***undertaken within the recommended timeframe*** (the last 12 months; Table S2). (DOCX 29 kb)

## Abbreviations

ACCHS: Aboriginal Community Controlled Health Service
BMI: Body Mass Index
DCP: Diabetes Care Plan
HA: Health Assessment
nKPI: National Key Performance Indicator
