# Supplementary material for: Concordance between self-report and medical records of preventive healthcare delivery among a sample of disadvantaged patients from four aboriginal community controlled health services
Source: BMC Health Serv Res. 2019 Feb 8;19:111. doi: 10.1186/s12913-019-3930-7 (PMC6368754; doi:10.1186/s12913-019-3930-7)
Supplement: Supplementary file 1 — Table S1. and Table S2. Description of data: Detailed concordance data (including agreement, disagreement and missing records) for comparison a) whether an item had ever been undertaken (Table S1) and for comparison b) whether an item had been undertaken within the recommended timeframe (the last 12 months; Table S2). (DOCX 29 kb) [file 12913_2019_3930_MOESM1_ESM.docx]

**Additional file 1:**

**Table S1: Self-report responses and medical record data for whether health care items had been assessed and the degree of concordance between self-report and medical records [data collected 2013-14 from NSW]**

| **Health care item** | **Self-report responses n (%)** | | | | **Medical record n (%)** | | | **Concordance for whether items had ever been assessed %** |
| --- | --- | --- | --- | --- | --- | --- | --- | --- |
|  | **Yes** | **No** | **Not sure** | **Missing** | **Yes** | **No** | **Missing** |  |
| BMI (n=82)^a^ | 80 (98%) | 0 | 2 (2%) | - | 80 (98%) | 2 (2%) | - | 95% |
| Waist circumference (n=82)^a^ | 52 (63%) | 16 (20%) | 14 (17%) | - | 61 (74%) | 21 (26%) | - | 67% |
| Blood pressure (n=109) | 101 (93%) | 0 | 7 (6%) | 1 (1%) | 109 (100%) | 0 | - | 93% |
| Smoking status (n=79)^b^ | 66 (84%) | 5 (6%) | 8 (10%) | - | 75 (95%) | 4 (5%) | - | 81% |
| Alcohol intake (n=109) | 71 (65%) | 22 (20%) | 15 (14%) | 1 (1%) | 94 (86%) | 15 (14%) | - | 55% |
| Physical activity (n=109) | 87 (80%) | 5 (5%) | 17 (16%) | - | 65 (60%) | 33 (30%) | 11 (10%) | 49% |
| Diet (n=109) | 91 (83%) | 6 (6%) | 11 (10%) | 1 (1%) | 74 (68%) | 25 (23%) | 10 (9%) | 62% |
| Health Check  [Medicare Item 715] (n=109) | 92 (84%) | 9 (8%) | 7 (6%) | 1 (1%) | 72 (66%) | 31 (28%) | 6 (6%) | 61% |
| Diabetes Care Plan [Medicare Item 721] (n=37)^c^ | 21 (57%) | 4 (11%) | 7 (19%) | 5 (14%) | 16 (43%) | 18 (49%) | 3 (8%) | 68% |

^a^Data for BMI and WC for one site was lost during data merging

^b^Includes only self-reported current and ex-smokers, as never smokers skipped the question about when they were last asked about their smoking status

^c^Those without diabetes (according to self-report) did not compete the DCP item and were therefore excluded from this analysis

**Table S2: Self-report responses and medical record data for whether health care items had been assessed within the recommended time frame (the last 12 months) and the degree of concordance between self-report and medical records [data collected 2013-14 from NSW]**

| **Health care item** | **Self-report responses n (%)** | | | | **Medical record n (%)** | | | **Concordance for whether items had been assessed within timeframe %** |
| --- | --- | --- | --- | --- | --- | --- | --- | --- |
|  | **Yes** | **No** | **Not sure** | **Missing** | **Yes** | **No** | **Missing** |  |
| BMI (n=82)^a^ | 73 (89%) | 7 (9%) | 2 (2%) | - | 77 (94%) | 3 (4%) | 2 (2%) | 83% |
| Waist circumference (n=82)^a^ | 45 (55%) | 23 (28%) | 14 (17%) | - | 59 (72%) | 23 (28%) | 0 | 59% |
| Blood pressure (n=109) | 100 (92%) | 1 (1%) | 7 (6%) | 1 (1%) | 108 (99%) | 0 | 1 (1%) | 92% |
| Smoking status (n=79)^b^ | 55 (70%) | 16 (20%) | 8 (10%) | - | 58 (73%) | 19 (24%) | 2 (3%) | 68% |
| Alcohol intake (n=109) | 61 (56%) | 32 (29%) | 15 (14%) | 1 (1%) | 70 (64%) | 35 (32%) | 4 (4%) | 53% |
| Physical activity (n=109) | 83 (76%) | 9 (8%) | 17 (16%) | - | 59 (54%) | 34 (31%) | 16 (15%) | 45% |
| Diet (n=109) | 86 (79%) | 11 (10%) | 11 (10%) | 1 (1%) | 61 (56%) | 29 (27%) | 19 (17%) | 55% |
| Health Check  [Medicare Item 715] (n=109) | 80 (73%) | 17 (16%) | 11 (10%) | 1 (1%) | 66 (61%) | 35 (32%) | 8 (7%) | 50% |
| Diabetes Care Plan [Medicare Item 721]** (n=37)^c^ | 11 (30%) | 14 (38%) | 7 (19%) | 5 (14%) | 13 (35%) | 21 (57%) | 3 (8%) | 68% |

^a^Data for BMI and WC for one site was lost during data merging

^b^Includes only self-reported current and ex-smokers, as never smokers skipped the question about when they were last asked about their smoking status

^c^Those without diabetes (according to self-report) did not compete the DCP item and were therefore excluded from this analysis
